# DUAL-TASK CHANGES IN PREFRONTAL ACTIVATION AND GAIT QUALITY IN OLDER ADULTS

**DOI:** 10.1093/geroni/igac059.1311

**Published:** 2022-12-20

**Authors:** Emma Baillargeon, Anisha Suri, Theodore Huppert, Ervin Sejdic, Andrea Rosso

**Affiliations:** University of Pittsburgh, Pittsburgh, Pennsylvania, United States; Swanson School of Engineering, University of Pittsburgh, Pittsburgh, Pennsylvania, United States; University of Pittsburgh, Pittsburgh, Pennsylvania, United States; University of Toronto, Toronto, Ontario, Canada; University of Pittsburgh, Pittsburgh, Pennsylvania, United States

## Abstract

We compared the impact of performing dual-task walking on gait quality and prefrontal cortical activation assessed by functional near-infrared spectroscopy (fNIRS). We hypothesized a greater increase in fNIRS averaged over the left prefrontal cortex during dual-task walking would be associated with a greater decrease in gait quality (increased step-time variability; decreased gait speed, cadence, smoothness, and adaptability). In older adults (n=60, 75±5.8 years, 57% female), we quantified the change in fNIRS and gait metrics from single-task walking (even surface) to walking with attentional (reciting every-other letter of the alphabet) and physical (uneven surface) dual-task challenges using four 15m repetitions of each task. Gait metrics were computed from a tri-axial accelerometer at the lower-back. Changes in fNIRS from single to dual-task walking were not associated with changes in gait quality for both attentional and physical challenges (Spearman correlations, all p>0.08). Variability in response across individuals may contribute to our findings.

